# Variants of *NOD2* in *Leishmania guyanensis*-infected patients with cutaneous leishmaniasis and correlations with plasma circulating pro-inflammatory cytokines

**DOI:** 10.1371/journal.pone.0281814

**Published:** 2023-02-16

**Authors:** Tirza Gabrielle Ramos de Mesquita, José do Espírito Santo Junior, Josué Lacerda de Souza, Lener Santos da Silva, Tuanny Arruda do Nascimento, Mara Lúcia Gomes de Souza, Marcus Vinitius de Farias Guerra, Rajendranath Ramasawmy

**Affiliations:** 1 Programa de Pós-Graduação em Medicina Tropical, Universidade do Estado do Amazonas, Manaus, Brazil; 2 Fundação de Medicina Tropical Doutor Heitor Vieira Dourado, Manaus, Brazil; 3 Programa de Pós-Graduação em Imunologia Básica e Aplicada, Instituto de Ciências Biológicas, Universidade Federal do Amazonas, Manaus, Amazonas, Brazil; 4 Faculdade de Medicina Nilton Lins, Universidade Nilton Lins, Manaus, Brazil; 5 Programa de Pós-Graduação em Biodiversidade e Biotecnologia da Amazonia Legal (Rede Bionorte), Universidade do Estado do Amazonas, Manaus, Brazil; 6 Genomic Health Surveillance Network: Optimization of Assistance and Research in The State of Amazonas – REGESAM, Manaus, Amazonas, Brazil; Universidade Federal da Bahia, BRAZIL

## Abstract

Leishmaniases, a group of vector-borne diseases, are caused by the protozoan intracellular parasite *Leishmania* (*L*.) and are transmitted by the phlebotomine sandflies. A wide range of clinical manifestations in *L*- infection is observed. The clinical outcome ranges from asymptomatic, cutaneous leishmaniasis (CL) to severe mucosal leishmaniasis (ML) or visceral leishmaniasis (VL), depending on the *L*. species. Interestingly, only a fraction of *L*.-infected individuals progress to disease development, suggesting a key role of host genetics in the clinical outcome. NOD2 plays a critical role in the control of host defense and inflammation. The NOD2-RIK2 pathway is involved in developing a Th1- type response in patients with VL and C57BL/6 mice infected with *L*. *infantum*. We investigated whether variants in the *NOD2* gene (R702W rs2066844, G908R rs2066845, and L1007fsinsC rs2066847) are associated with susceptibility to CL caused by *L*. *guyanensis (Lg)* in 837 patients with *Lg*-Cl and 797 healthy controls (HC) with no history of leishmaniasis. Both patients and HC are from the same endemic area of the Amazonas state of Brazil. The variants R702W and G908R were genotyped by polymerase chain reaction-restriction fragment length polymorphism (PCR-RFLP), and L1007fsinsC was by direct nucleotide sequencing. The minor allele frequency (MAF) of L1007fsinsC was 0.5% among the patients with *Lg*-CL and 0.6% in the healthy controls group. R702W genotypes frequencies were similar in both groups. Only 1% and 1.6% were heterozygous for G908R among the patients with *Lg*-CL and HC, respectively. None of the variants revealed any association with susceptibility to the development of *Lg*-CL. Correlations of genotypes with the level of plasma cytokines revealed that individuals with the mutant alleles of R702W tend to have low levels of IFN-γ. G908R heterozygotes also tend to have low IFN-γ, TNF-α, IL-17, and IL-8. Variants of *NOD2* are not involved in the pathogenesis of *Lg-*CL.

## Introduction

Leishmaniases are vector-borne parasitic diseases caused by the protozoan *Leishmania* (*L*.), are transmitted by phlebotomine sandflies. Leishmaniases manifest into visceral and tegumentary leishmaniasis. Tegumentary leishmaniasis may manifest as localized cutaneous leishmaniasis (CL), diffuse CL, disseminated CL, and mucocutaneous leishmaniasis (ML). Symptoms of visceral leishmaniasis (VL), a life-threatening disease and fatal in 95% of untreated cases, manifest by irregular bouts of fever, weight loss, enlargement of the spleen and liver, and anemia. Nearly one billion individuals are at risk of developing leishmaniases, which are present in more than 98 countries [1].

Leishmaniases are endemic in 97 countries and affect around 12 million people worldwide, especially in the tropic and subtropical countries. According to the world health organization, 253,435 cases of CL were notified in 2018 [1]. In Brazil, 16,813 new cases of CL were reported [2]. In the Amazonas, 1692 new cases of CL were registered [2].

VL is caused by *L*. *infantum* in America, Central Asia, and the Middle East, and by *L*. *donovani* in Asia and Africa. In Brazil, *L*. *braziliensis*, *L*. *guyanensis*, *L*. *lainsoni*, *L*. *amazonensis*, *L*. *shawi*, *L*. *naiffi*, and *L*. *lindenberghi* are the major species causing American tegumentary leishmaniasis. *L*. *guyanensis* is the main etiological agent of CL in the Amazonas [3].

NOD-like receptors (NLR) induce host protective immunity against intracellular parasitic infections [4, 5]. NOD2, also known as CARD15, triggers host-innate immunity [6]. NOD2 recognizes conserved muramyl dipeptides of bacterial peptidoglycan [7, 8] and activates nuclear factor-kappaB (NF-ĸB) through the RIP2 kinase pathway [9, 10], to trigger the transcription of proinflammatory cytokines such as IL-12 and IL-1β [11, 12]. Th1 cell immune response protects against *L*.-infection by releasing proinflammatory cytokines, IL-12, IFN-γ, and TNF-α [13].

Peripheral blood mononuclear cells (PBMCs) of individuals bearing the loss of function genetic variants of *NOD2* displayed lower levels of proinflammatory cytokines than individuals with the functional variants when exposed to *L*. *amazonensis* or *L*. *braziliensis in vitro* [14]. The NOD2-RIK2 pathway is involved in the development of a Th1- type response in patients with VL and C57BL/6 mice infected with *L*. *infantum* [15]. *Leishmania* possesses lipophosphoglycans (LPG), glycoinositolphospholipids (GIPLs), and glycoprotein 63 (gp63) in its membrane which are recognized by pathogen recognition receptors [16, 17].

LPG can activate the NOD-like receptor NLPR3 in a non-canonical pathway [18]. RIPK2 is a critical kinase for the NOD2 signaling cascade and production of inflammatory cytokines. Inhibition of RIPK2 phosphorylation in PBMCs, exposed to LPG of *L*. *braziliensis* or *L*. *amazonensis*, leads to a reduction of IL-32, IL-6, and IL-1β [19].

*NOD2* is on chromosome 16q21. Loss of function variants in the *NOD2* gene (R702W, G908R, and L1007fsinsC) leads to a hypoimmune response and impairs NOD2 activation [7, 20]. The missense mutation rs2066844 C/T (R702W), located in exon 4, substitutes arginine at codon position 702 with tryptophan. The rs2066845 C/G (G908R) in exon 8 leads to the substitution of glycine at position 908 with arginine, while the rs2066847 (L1007fsinsC), located in exon 11, inserts a cytosine (C) at nucleotide position 3020 leading to a frameshift and premature stop codon at codon 1007. All these variants are present in the leucine-rich region of the protein that is involved in ligand recognition [21].

Interestingly, the clinical manifestations of infection with *Leishmania* depend on the host-genetic background, the vector, the site of infection, the skin microbiota, and the *Leishmania* spp [22, 23]. In light of the importance of NOD2 in immune response, this study evaluated whether the genetic variants (R702W, G908R, and L1007fsinsC) of *NOD2* may be associated with susceptibility to CL caused by *L*. *guyanensis* and influenced plasma circulating proinflammatory cytokines.

## Materials and methods

This case-control study was performed according to the guidelines strengthening the reporting of genetic association studies (STREGA). The study was carried out in the state of Amazonas, Brazil. Patients with CL as well as healthy controls with no scar or history of leishmaniasis came from the peripheral regions of Manaus, the capital city of Amazonas. Briefly, the areas surrounding BR-174 and AM-010 in the peripheral regions of Manaus became endemicity for *Lg*-infection due to human invasion (i.e., communities of Pau-Rosa, Cooperativa, Agua-Branca, Leão, and Brasileirinho). Patients with CL were attended at the Fundação de Medicina Tropical Doutor Heitor Vieira Dourado (FMT-HVD), the referral centre for treatment of leishmaniasis.

### Ethical approval

This study was carried out under the Helsinki Declaration and was approved by the Research Ethics Committee of the FMT-HVD/2012 (CAAE—Certificado de Apresentacão para Apreciacão Etica: 09995212.0.0000.0005). All volunteers provided written informed consent for sample collection and subsequent analysis. For patients younger than 18 years of age, the parents or responsible party consented to the child’s participation and provided the written Informed Consent Form.

### Identification of *Leishmania spp*.

Patients with CL provided a biopsy specimen of the skin lesion to identify the *Leishmania* species. DNA was extracted from the biopsies and submitted to *Leishmania viannia* subgenus-specific PCR according to the established protocols [24, 25]. *Leishmania spp*. was identified by direct nucleotide sequencing of a fragment of HSP 70 and mini-exon genes as described elsewhere [26].

### DNA extraction from whole blood for alleles discrimination of variants of NOD2

All the participants gave five mL of peripheral blood by venipuncture in a vacutainer tube containing ethylenediaminetetraacetatic acid (Becton Dickinson). Plasma was separated and kept frozen in a freezer at -80°C until usage. Genomic DNA was extracted by the salting out method [27].

The variant L1007fsinsC was genotyped by direct nucleotide sequencing. The following pair of primers, forward 5`-GGATGTGTCTAAGGGACAGGTG-3`and reverse 5`-CTGAGGTTCGGAGAGCTA-3`were designed to amplify a fragment of 251bp. Nucleotide sequencing was performed using the forward primer with the kit BigDyes from Applied Biosystem (Thermofisher, MA USA) following the protocols suggested by the manufacturer.

The variants R702W (rs2066844 C/T) and G908R (rs2066845 G/C) of the *NOD2* gene were typed by PCR-RFLP using the restriction enzymes Hpa II for R702W and Hha I for G908R (New England Biolabs). The generated PCR fragment was 171bp for R702W, using the following pair of primers, forward 5`- GCACAACCTTCAGATCACAGCA-3`and reverse 5`- GCTGGCGGGATGGAGTGGAAG-3`. 10uL of the PCR products was digested with the restriction enzymes Hpa II. The PCR product (171bp) is cleaved into three fragments, 71bp, 54bp, and 46bp in the presence of allele C and in two fragments (125bp and 46bp) when the T allele is present. A fragment of 249 bp was amplified by PCR using the following pair of primers, forward 5`-CAGTGAGGCCACTCTGGGATTG-3`and reverse 5`-AAAACTGCAGGATAGACTCT-3`for G908R. If the allele G is present, the 245bp fragment remains uncleaved by the restriction enzyme Hha I, while in the presence of allele C, the fragment is cleaved into 145 and 104 bp. PCR restriction fragments were size separated by electrophoresis in 3% agarose gel.

### Cytokine assay by Luminex

5 mL of blood from patients with *Lg*-CL before antimonial treatment and from healthy controls were collected. Plasma was separated and kept at -80°C until plasma cytokines assay.

The levels of IFNγ, IL-1β, IL-6, IL-8, IL-12 (p70), IL-13, IL-17A, RANTES, and TNFα were determined using the multiplex cytokine commercial kit Bio-PlexPro-Human Cytokine GrpI Panel 27-Plex (Bio-Rad) according to the manufacturer’s instructions in the Bio-Plex 200 Protein Array System (Luminex Corporation).

### Statistical analysis

Genotypes and allele frequencies were calculated by direct counting. HWE (Hardy-Weinberg Equilibrium) and logistic regression analysis with a confidence interval (CI) of 95% and χ^2^ test to compare patients with *Lg*-Cl and healthy control groups (HC) were performed using the website https://ihg.helmholtz-muenchen.de/cgi-bin/hw/hwa1.pl. Quantitative trait analysis was used to correlate the *NOD2* genotypes with circulating plasma cytokines in R software (version 4.2.1) using package SNPassoc (version 2.0–11) and ggplot2 package for visualization. P values of cytokines correlations with genotypes were corrected by the false discovery rate (FDR) of Benjamini-Hochberg.

## Results

### Population of the study

The study population was previously described [28, 29]. This study includes 850 patients with *Lg*-CL and 891 healthy controls (HC). The HC has the same socio-epidemiology characteristics as the patients and were from the same endemic area. The patients with *Lg*-CL were first-time diagnosed, treatment-naïve, and had fewer or equal to six lesions. The HC was not stratified as asymptomatic as delayed- test of hypersensitivity to *Leishmania*-antigens was not performed. All the participants were HIV-negative and devoid of cardiac, renal, or diabetes disease. Most of the participants are agricultural or farms-workers. The basic characteristics of the study population are shown in (Table 1).

**Table 1:** Basic characteristics of the patients with *Leishmania guyanensis*-cutaneous leishmaniasis (*Lg*-CL) and healthy controls (HC).

Patients with *Lg*-CL			HC		
	N = 850		N = 891		*p*-value ^1^
	Males	Females	Males	Females	
**Sex**	639 (75%)	211 (25%)	608 (68%)	283 (32%)	0.001
**Age (mean ± SEM** ^2^**) years**	34.4 ± 13.7	37.5 ± 15.7	42 ± 17.5	40 ± 17.4	

Abbreviations:

^1^ *p*-value <0.05 is significant,

^2^ SEM: Standard error of mean.

The HC is slightly older than the patients with *Lg*-CL (P<0.0001). The age of male participants was higher in the HC group to male patients with *Lg*-CL (P<0.0001). There was no age difference among female participants (P<0.077).

The variants R702W, G908R, and L1007fsinsC were genotyped in 821, 837, and 762 patients with *Lg*-CL, respectively. Similarly, the R702W, G908R, and L1007fsinsC were genotyped in 777, 797, and 746 healthy controls, respectively. The distribution of the genotypes of the three variants was in Hardy-Weinberg equilibrium in both groups. The frequencies of the genotypes and alleles are shown in (Table 2).

**Table 2:** Genotypes and alleles frequencies of the variants R702W, G908R, and L1007fsinsC of the *NOD2* gene among patients with *Lg*-CL (cases) and healthy controls (HC).

Genotypes		Cases (%)	HC (%)	Comparisons	*P*-value ^1^	OR ^2^(95% CI^3^)
		n = 821	n = 777			
**R702W**	C/C	789 (96,1)	748 (96,3)	CC vs. TT	0.96	0.94 [0.06–15]
**rs2066844**	C/T	31 (3,8)	28 (3,6)	CC vs. CT	0.85	1.0 [0.6–1.7]
	T/T	1 (0,1)	1 (0,1)	CC vs CT+TT	0.86	1.0 [0.6–1.7]
**Alleles**	C	1609 (98)	1524 (98)	C vs. T	0.87	1.0 [0.6–1.7]
	T	33 (2)	30 (2)			
		**n = 837**	**n = 797**			
**G908R rs2066845**	G/G	828 (98,9)	784 (98,4)	GG vs CC	0.33	2.8 [0.1–69]
	G/C	8 (1)	13 (1,6)	GG vs. GC	0.22	0.58 [0.2–1.4]
	C/C	1 (0,1)	0	GG vs GC+CC	0.32	0.65 [0.2–1.5]
**Alleles**	G	1664 (99,4)	1581 (99,2)	C vs. G	0.45	0.73 [0.3–1.6]
	C	10 (0,6)	13 (0,8)			
		**n = 762**	**n = 746**			
**L1007fsinsC rs2066847**	-/-	754 (99)	737 (99)			
	-/C	8 (1)	9 (1)	-/- vs. -/C	0.77	0.86 [0.3–2.2]
	C/C	0	0	- vs. C	0.77	0.87 [0.33–2.2]
**Alleles**	-	1516 (99,5)	1483 (99,4)			
	C	8 (0,5)	9 (0,6)			

Abbreviations:

^1^ p-value <0.05 is significant,

^2^ OR: Odds ratio.

^3^ CI: confidence interval.

The minus sign–of the L1007fsinsC denotes the absence of the insertion of the nucleotide cytosine C.

For the frameshift variant L1007fs, the minor allele frequency (MAF) was 0.5% among the patients with *Lg*-CL and 0.6% in the HC group. Only eight individuals (1%) among the patients with *Lg*-CL and nine (1%) among the HC were heterozygotes for the L1007fs variant. For the R702W, 31 (3.8%) and 28(3.6%) were heterozygotes in the patients and HC groups, respectively. Similarly, only eight (1%) and 13 (1.6%) individuals were heterozygotes for G908R among the patients with *Lg*-CL and HC, respectively. None of the variants revealed any association with either susceptibility or protection to *Lg*-CL. (Table 3) showed the statistical comparisons between the patients with *Lg*-CL and HC according to inheritance models and revealed no association with susceptibility to *Lg*-CL.

**Table 3:** Statistical comparisons between patients with *Lg*-CL and healthy controls based on inheritance models with p-values and odd ratios adjusted for age and sex.

	Inheritance models		*p-*value	*Padj* ^4^
			[OR (95% CI]	[OR ^5^ *adj* (95% CI ^6^]
**R702W rs2066844**	1	C/C vs C/T+T/T	0.86 [1.05 (0.63–1.75)]	0.96 [1.01 (0.59–1.72)]
	2	C/C + C/T vs T/T	0.96 [0.95 (0.06–15.16)]	0.77 [0.66 (0.04–11.54)]
	3	C/C+T/T vs CT	0.85 [1.05 (0.62–1.77)]	0.92 [1.03 (0.60–1.77)]
**G908R**	1	C/C vs C/G+G/G	0.32 [0.66 (0.28–1.54)]	0.40 [1.45(0.61–3.46)]
	2	C/C+C/G vs G/G	1.00 [0.00 (0.00–0.00)]	0.33 [1.59 (0.00–0.00)]
**rs2066845**	3	C/C+G/G vs C/G	0.22 [0.58 (0.24–1.41)]	0.30 [1.60(0.65–3.93)]
**L1007fsinsC rs2066847**		-/C vs C/C	0.77 [0.87 (0.33–2.26)]	0.6 [1.31 (0.48–3.56)]

Abbreviations:

^1^: dominant,

^2^: recessive, and

^3^: overdominant.

^4^ *Padj*: *p*-value adjusted by sex and age,

^5^ OR: odds ratio,

^6^ CI: confidence interval.

The minus sign–of the L1007fsinsC denotes the absence of the insertion of the nucleotide cytosine C.

None of the participants had more than one of the three variants. The minor alleles of the three variants (R702W, G908R, and L1007fsinsC) termed allele O and wild-type alleles as A were statistically compared between the patients with *Lg*-CL and HC (Table 4). Heterozygotes A/O frequency was 6.3% and 5.6% among the healthy controls and patients with *Lg*-CL, respectively. The frequency of the minor allele O was 3.3% among the HC and 3.1% in the patients with *Lg*-CL. Comparisons of genotype or allele frequencies between patients with *Lg*-CL and healthy controls did not reveal any association. The minor alleles were not associated with either susceptibility or protection to *Lg*-CL.

**Table 4:** Pooled combination of the genotypes and alleles of the three variants R702W, G908R, and L1007fsinsC among patients with *Lg*-CL (cases) and healthy controls (HC).

Genotypes		Cases (%)	HC (%)	Comparisons	*p*-value ^1^	OR ^2^(95% CI^3^)
		n = 837	n = 797			
***NOD2***	A/A	788 (94,1)	746 (93,6)	A/A vs. O/O	0.60	1.8 [0.1–21]
	A/O	47 (5,6)	50 (6,3)	A/A vs. A/O	0.57	0.9 [0.6–1.3]
	O/O	2 (0,3)	1 (0,1)	A/A vs. A/O + O/O	0.65	0.9 [0.6–13]
**Alleles**	O	1623 (96,9)	1542 (96,7)	A vs. O	0.72	0.9 [0.6–1.4]
	A	51 (3,1)	52 (3,3)			

Abbreviations: A represents the pooled wild-type alleles and O the pooled mutant alleles of R702W, G908R, and L1007fsinsC.

^1^ p-value <0.05 is significant;

^2^ OR: odds ratio;

^3^ CI: confidence interval.

### Plasma cytokines levels by NOD2 genotypes

Assay of plasma cytokines levels was in 354 patients with *Lg-*CL (264 males and 90 females) and 376 (269 males and 107 females) HC. The mean age (mean ± standard error of the mean) of the male patients with *Lg-*CL and HC were 39.8±1.57 and 45.2±1.58 years old, respectively. Similarly, female patients with *Lg-*CL and HC were 34.6±0.80 and 43.7±1.80 years old, respectively.

**Fig 1:**
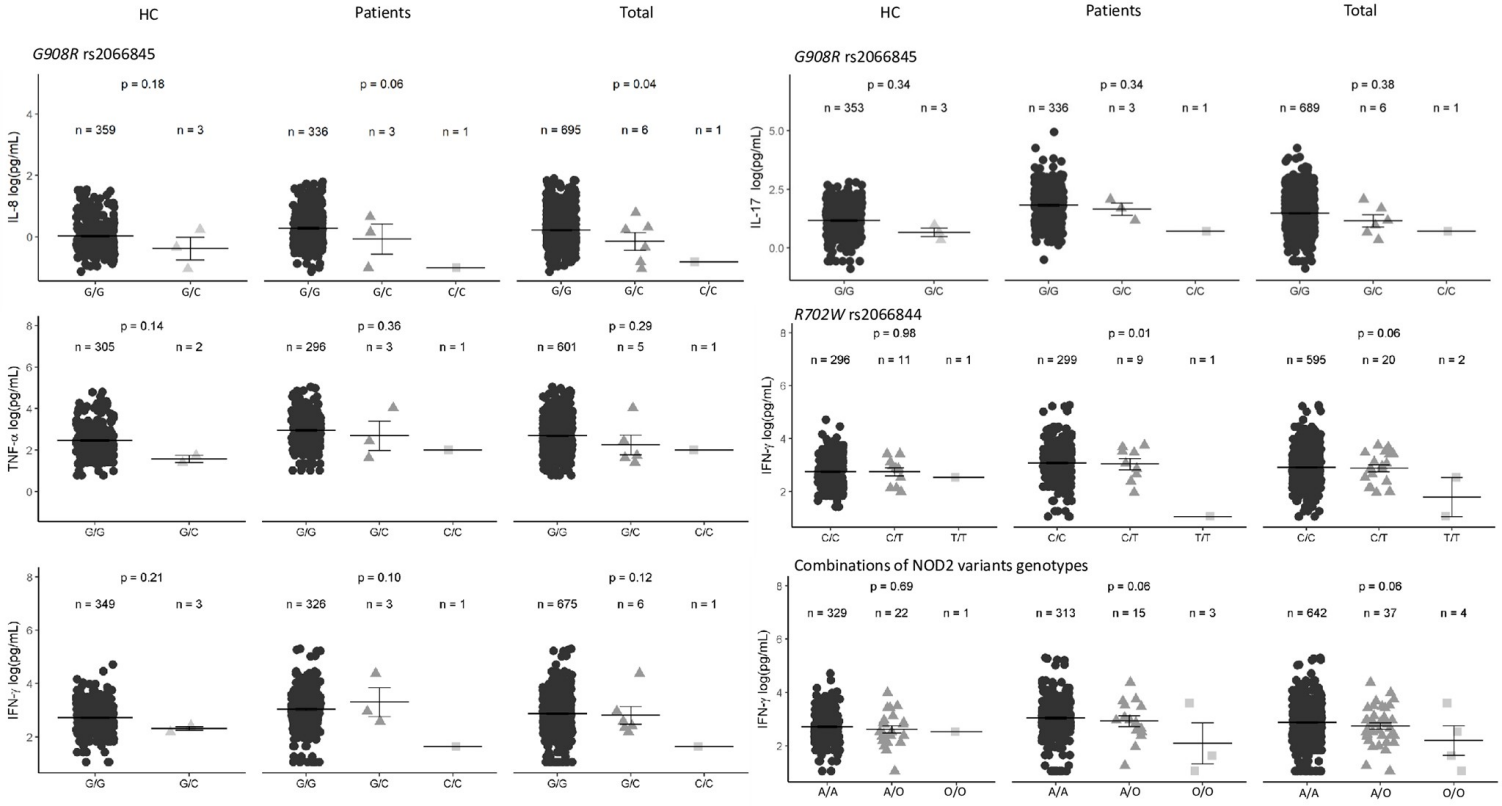
Evaluation of the *NOD2* variants genotypes on circulating IL-8, IL-17, IFN-γ, and TNF-α plasma levels in HC, patients with CL, and total (HC + patients with *Lg*- CL) adjusted for age and sex. The combination for all genotypes, common homozygous (A/A), heterozygous (A/O) and rare homozygous (O/O) are also shown in HC, Patients, and Total (A represent wild-type alleles and O mutant alleles of the three variants). The crossbars (black) represent the mean concentrations in picogram per milliliter log-scale transformed [log(pg/mL)] and the error bars represent the standard error (SE) of means. P values < 0.05 are considered significant. P values were corrected for False Discovery Rates using Benjamini Hochberg procedures.

Monocytes and lymphocytes from individuals harboring the L1007fs, infected with either *L*. *braziliensis* or *L*. *amazonensis*, exhibit lower levels of TNFα, IL-1β, IL-6, IL-8, IFN-γ, and IL-17 [14]. Genotypes of the three variants were evaluated with levels of plasma cytokines to investigate whether they may influence the levels of proinflammatory cytokines, as shown in (S1–S3 Figs). A pooled analysis of all genotypes of the three variants is shown in S4 Fig. G908R genotypes tended to correlate with plasma cytokine TNF-α, IFN-γ, IL-17, and the chemokine IL-8, but did not reach statistical significance (Fig 1).

The mean levels, expressed in log (pg/mL), of plasma circulating IL-8, IL-17, IFN-γ, and TNF-α by genotypes of the three variants, are shown in (S1 Table). Individuals heterozygous for the variant G908R tended to correlate with low levels of IL-8 among the HC (p = 0.18) and in patients with *Lg*-CL (p = 0.06). Combining patients and HC, heterozygous individuals G/C have a mean of plasma IL-8 of G/C = -0.14±0.29 log(pg/mL) compared to individuals G/G = 0.21±0.02 log(pg/mL) and C/C = -0.82±0.0 log(pg/mL), p = 0.04). Of note, after P values correction according to False Discovery Rates following Benjamini and Hochberg, the statistical significance is abolished. Similarly, heterozygous individuals tended to have low plasma TNF-α, IL-17, and IFN-γ mean levels.

The genotypes of *NOD2* R702W seem to influence the plasma level of IFN-γ in both patients with *Lg*-CL and HC. Carriers of the C/C genotypes among patients with *Lg*-CL have higher mean levels than bearers of the C/T and T/T genotypes (p = 0.01). After corrections of P values by FDR, the statistical significance disappeared.

We also compared all individuals harboring at least one of the variants (heterozygotes or homozygotes for R702W, G908R, and L1007fsinsC mutant alleles) with the wild-type genotypes. Individuals with the mutant alleles seem to have lower levels of IFN-γ.

## Discussion

In endemic areas of leishmaniasis, only a fraction of the endemic population develops the disease. Discovering the exact cause that leads some individuals to progress to disease development in the endemic area while others sharing the same area of endemicity remain asymptomatic is challenging. Assessing the impact of genetic variation on immune gene expression and function may provide clues to the underlying mechanism of why some individuals are prone to develop the disease.

Monocytes isolated from patients with Crohn`s disease and bearing the R702W variant showed impaired activation of NF-kB signaling [30]. The variant R702W abrogates NF-kB activation when introduced into the HEK293 cell line, exposed to *Helicobacter pylori* [31]. In contrast, mice carrying a variant corresponding to the human L1007fsinsC displayed elevated NF-kB activation [32].

The NOD signaling pathway is involved in the pathogenesis of Crohn`s disease [20, 33]. Patients, carriers of deleterious variants of the *NOD2* gene, have low expression of NOD2 and increased expression of nuclear factor kappa B inhibitor alfa (NFKBIA) [34]. Furthermore, the same group of researchers compared patients bearing at least one of the variants R702W, G908R, or 1007fs to non-bearers and observed a significantly reduced NOD2 transcript count among the bearers of variants [34], allowing the researchers to suggest that genetic variation in the NOD2 pathway signaling culminates to a decreased proinflammatory response [34]. The frameshift variant L1007fs truncates most of the terminal of the leucine-rich region of NOD2 that is responsible for the recognition of LPS and leads to decreased NF-kB-activation [30].

Another study also showed that R702W, G908R, and L1007fs lead to decreased activity of NF-kB [7]. *NOD2* and *RIP2* knockout mice infected with *L*. *infantum* showed a higher parasite load and low IFNγ than wild-type mice [15]. Furthermore, RNAseq of peripheral blood from patients with VL revealed an upregulation of TH1 genes. A NOD2-driven Th1 response protects against *L*. *infantum* pathogenesis [15], *Trypanosoma cruzi* [35], and *Toxoplasma gondii* [36]. *NOD2* variants are associated with a high level of IL-17 in PBMC from patients with multiple sclerosis or with ocular toxoplasmosis stimulated with myelin-basic protein and soluble Toxoplasma antigen, respectively [37, 38].

Monocytes and lymphocytes from individuals harboring the L1007fs, infected with either *L*. *braziliensis* or *L*. *amazonensis*, exhibit lower levels of TNFα, IL-1β, IL-6, IL-8, IFN-γ, and IL-17 [14]. The genotypes of the three variants with levels of plasma cytokine were evaluated. *NOD2* G908R genotypes tended to correlate with plasma cytokine TNF-α, IFN-γ, IL-17, and the chemokine IL-8 was observed but did not reach statistical significance. The *NOD2* R702W genotypes showed a tendency of correlation with IFN-γ level. We compared all individuals harboring at least one of the variants with the wild-type genotypes. Carriers of the loss of function alleles tend to correlate with lower levels of IFN-γ compared to individuals homozygous for wild-type alleles. It is noteworthy to highlight that it is difficult *in vivo* to associate the exact impact of these variants on plasmatic cytokine levels as other genetic variants downstream in the NOD2 pathway may also contribute to the cytokines level compared to study *in vitro* by transfecting cell lines with the variant of interest.

Variants of the *NOD2* gene were initially associated with an increased risk of Crohn`s disease [21]. Since then, various studies have shown that the *NOD2* variants are associated with other diseases, including Blau syndrome [39], bipolar disorder [40], leprosy [41], and different types of cancer [42, 43]. NOD2 involvement in host defense has been shown extensively in cellular-system *in vitro*, animal models and genetic susceptibility studies in humans [44, 45]. We tested the three loss of function variants located in the leucine-rich region of the protein of NOD2 in a large sample of patients with *Lg*-Cl. None of the variants are associated with either susceptibility or protection to the development of CL.

In this study, the minor allele frequency (MAF) of the variant G908R was 0.8% and 0.6% among the healthy controls and patients with *Lg*-CL, respectively. For the R702W, similar frequencies were observed among healthy controls and patients. The MAF was 2%. Similarly, the MAF of 3020insC was 0.6% and 0.5% among healthy controls and patients, respectively. In the Caucasian population, the MAF of R702W is 4–5%, G908R 1–2%, and L1007fs 2–3% [46]. According to the SNP database of the NCBI (ncbi.nlm.nih.gov/snp), the MAF of G908R in European is 1.5%, African 0.3%, and Latin American 1.6%. The MAF for R702W in European is 4.6%, African 0.8%, and Latin American 2.5%. Similarly, the MAF for L1007fs in European is 9.4%, African 0.5%, and Latin American 0.6%. Notably, the population of this study is an admixture of Native American (50–60%), European (40–60%), and African ancestry (10%) [47].

R702W, G908R, and L1007fsinsC are genetically associated with Crohn`s disease in Europe and America, albeit not all individuals harboring the variants develop the disease [21, 48, 49]. In this study, none of the participants had Crohn`s disease. Furthermore, none of the participants was homozygote for the protein-truncating L100fsinsC.

To the best of our knowledge, this is the first study investigating the variants of NOD2 among patients with *Lg*-CL. Despite elegant experiments showing that NOD2 may recognize *Leishmania* components [14] and influence the expression of proinflammatory cytokines *in vitro*, there is no clear evidence that variants of NOD2 participate in the immunopathogenesis of *Lg*-CL. It is noteworthy to specify that various factors are involved in the development of CL. Any immune response genes also may play a role. The contribution of any gene may be small and sometimes difficult to detect when the frequencies of MAF are very low and may lack the power to observe any association.

These data demonstrate that variants of *NOD2* associated with decreased proinflammatory gene transcription are neither associated with protection nor susceptibility to *Lg*-CL. However, further studies in geographically distinct population groups of patients with *Lg*-CL or with CL caused by other species of *Leishmania* are needed before excluding genetic variants of NOD2 in the involvement of CL.

## Supporting information

S1 Fig NOD2 R702W (rs rs2066844) genotypes on circulating IL-1β, IL-6, IL-8, IL-17, IFN-γ, and TNF-α plasma levels in HC, Patients with CL and Total (HC + Patients with CL) adjusted by age and sex. The crossbars (black) represent the mean concentrations in picogram per milliliter log-scale transformed [log(pg/mL)] and the error bars represent the standard error (SE) of means. P values < 0.05 are considered significant.
(PDF)

S2 Fig NOD2 G908R (rs2066845) genotypes on circulating IL-1β, IL-6, IL-8, IL-17, IFN-γ, and TNF-α plasma levels in HC, Patients with CL and Total (HC + Patients with CL) adjusted by age and sex. The crossbars (black) represent the mean concentrations in picogram per milliliter log-scale transformed [log(pg/mL)] and the error bars represent the standard error (SE) of means. P values < 0.05 are considered significant.
(PDF)

S3 Fig NOD2 L1007fsinsC (rs2066847) genotypes on circulating IL-1β, IL-6, IL-8, IL-17, IFN-γ, and TNF-α plasma levels in HC, Patients with CL, and Total (HC + Patients with CL) adjusted by age and sex. The crossbars (black) represent the mean concentrations in picogram per milliliter log-scale transformed [log(pg/mL)] and the error bars represent the standard error (SE) of means. P values < 0.05 are considered significant.
(PDF)

S4 Fig Combinations of the three variants genotypes on circulating IL-1β, IL-6, IL-8, IL-17, IFN-γ, and TNF-α plasma levels in HC, Patients with CL, and Total (HC + Patients with CL) adjusted by age and sex. The crossbars (black) represent the mean concentrations in picogram per milliliter log-scale transformed [log(pg/mL)] and the error bars represent the standard error (SE) of means. P values < 0.05 are considered significant. The combination for all genotypes (G908R, R702W, and Lf1007ins C), common homozygous (A/A), heterozygous (A/O) and rare homozygous (O/O) are also shown in HC, Patients, and Total (A represent wild-type alleles and O mutant alleles of the three variants.
(PDF)

S1 Table Mean levels (mean±sem log pg/mL) of plasma circulating IL-8, TNF-α, IFN-γ, and IL-17 among patients with *Lg*-CL, HC, and totals (HC + Patients with *Lg*-CL) according to G908R, R702W, and pooled combinations of all genotypes. (DOCX)
