# Supplementary figures and images for: Variants of NOD2 in Leishmania guyanensis-infected patients with cutaneous leishmaniasis and correlations with plasma circulating pro-inflammatory cytokines
Source: PLoS One. 2023 Feb 16;18(2):e0281814. doi: 10.1371/journal.pone.0281814 (PMC9934361; doi:10.1371/journal.pone.0281814)

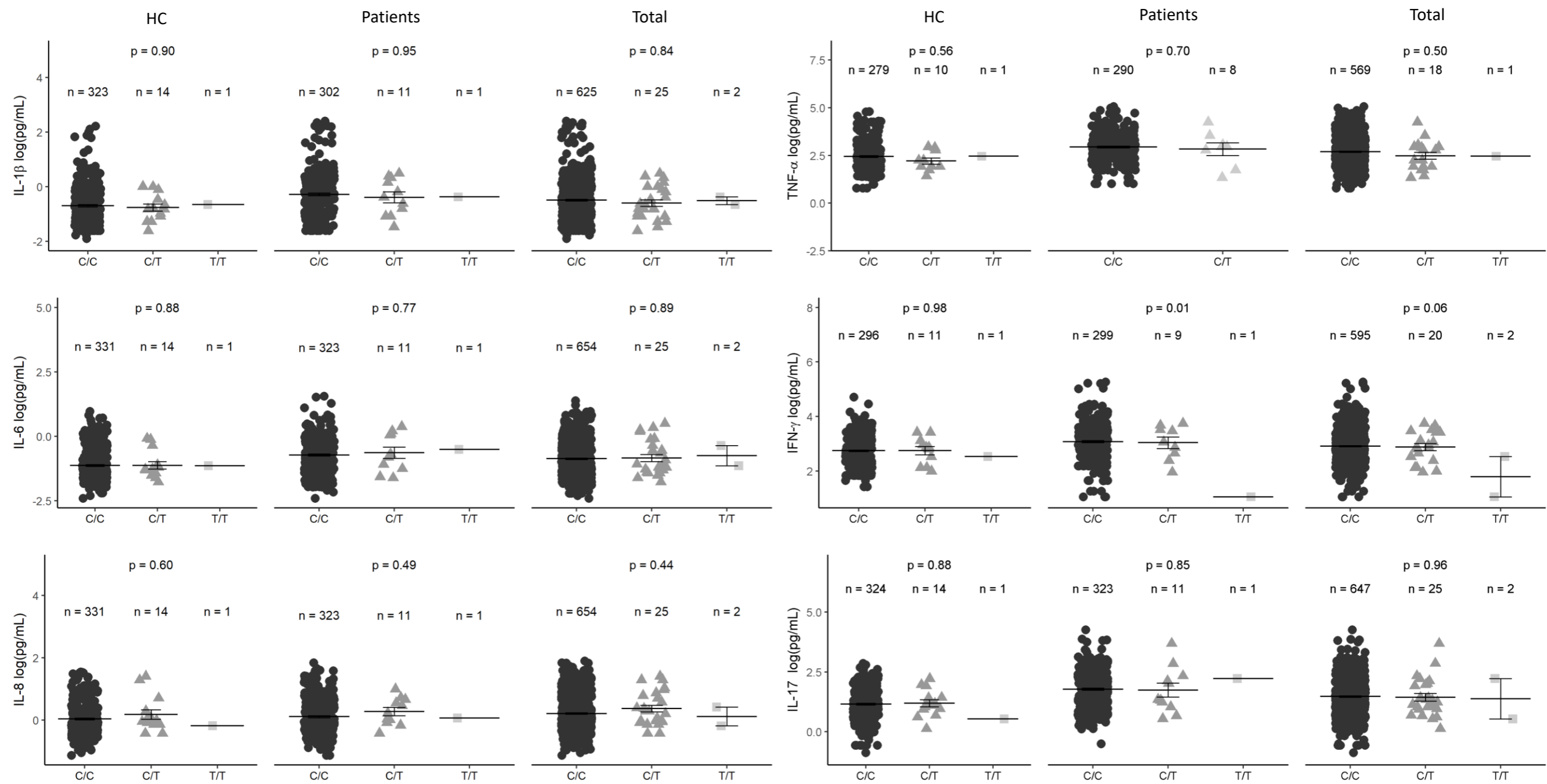

Supplement: S1 Fig — The crossbars (black) represent the mean concentrations in picogram per milliliter log-scale transformed [log(pg/mL)] and the error bars represent the standard error (SE) of means. P values < 0.05 are considered significant. (PDF) [file pone.0281814.s001.pdf]

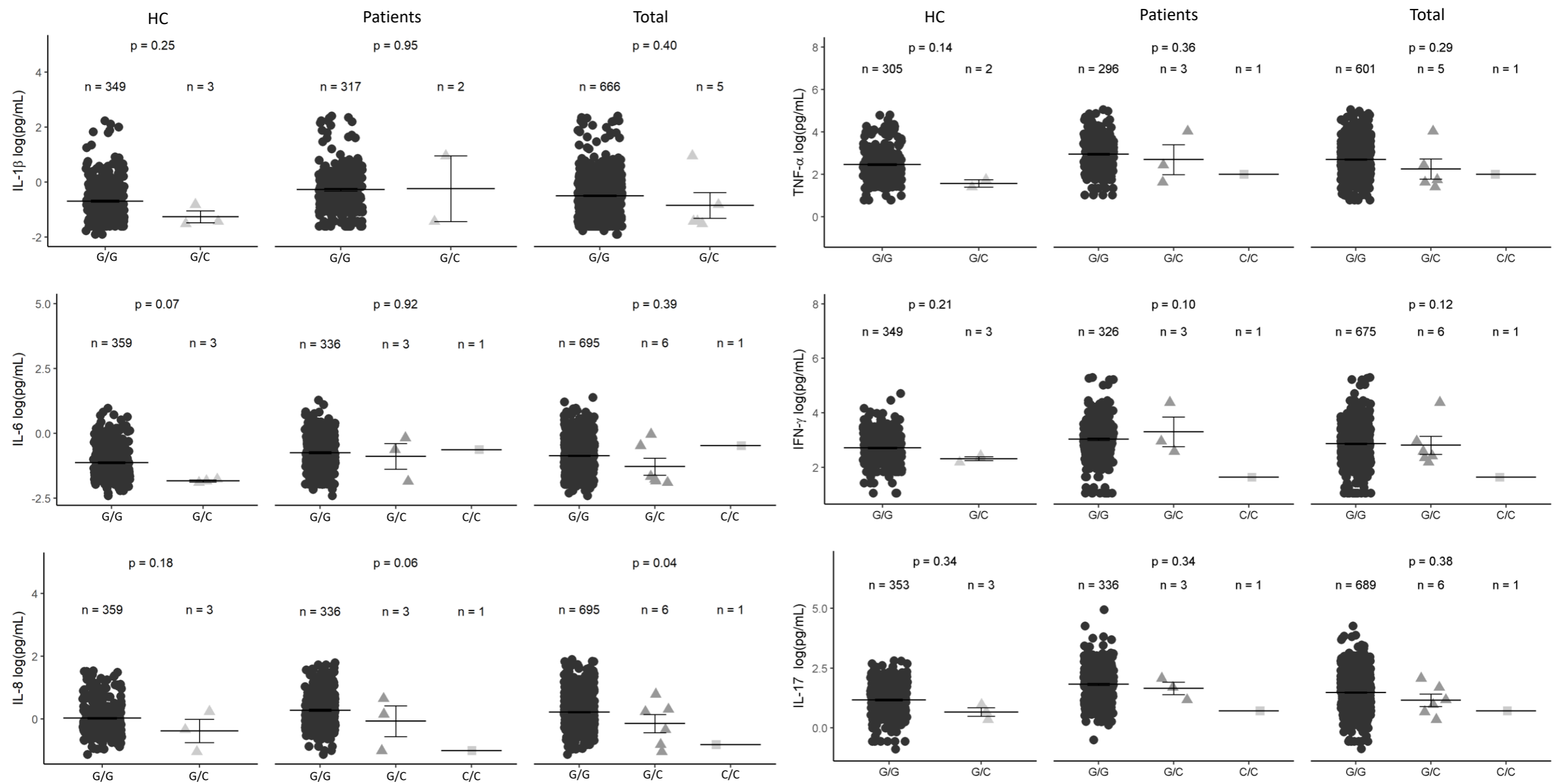

Supplement: S2 Fig — The crossbars (black) represent the mean concentrations in picogram per milliliter log-scale transformed [log(pg/mL)] and the error bars represent the standard error (SE) of means. P values < 0.05 are considered significant. (PDF) [file pone.0281814.s002.pdf]

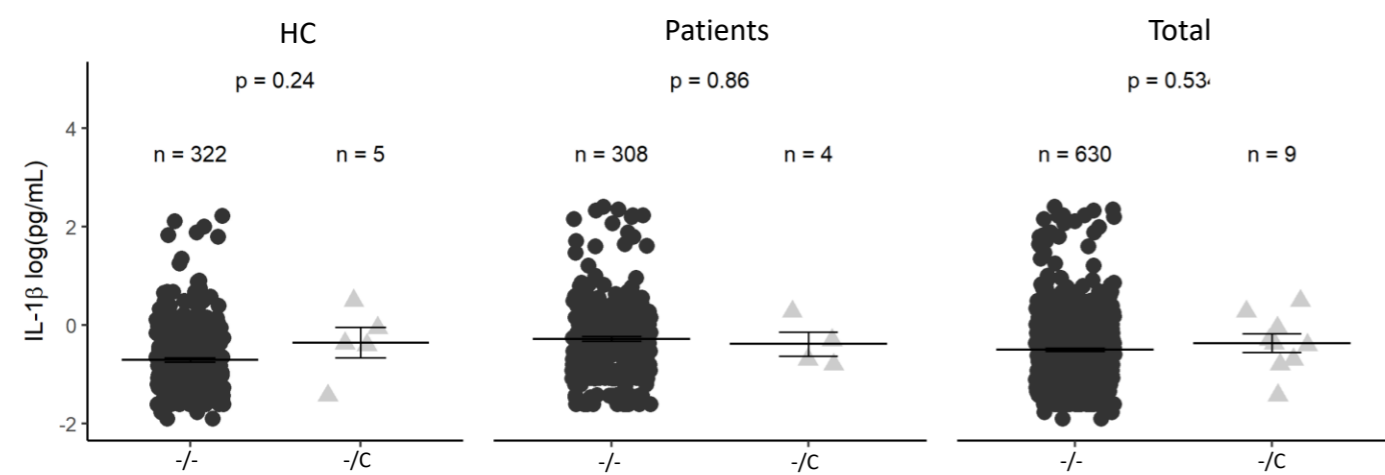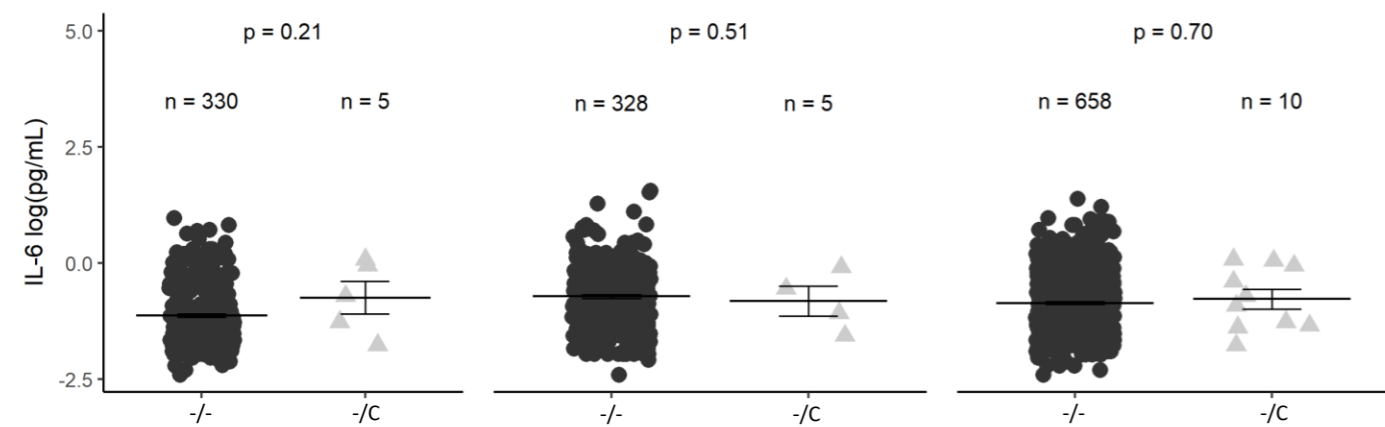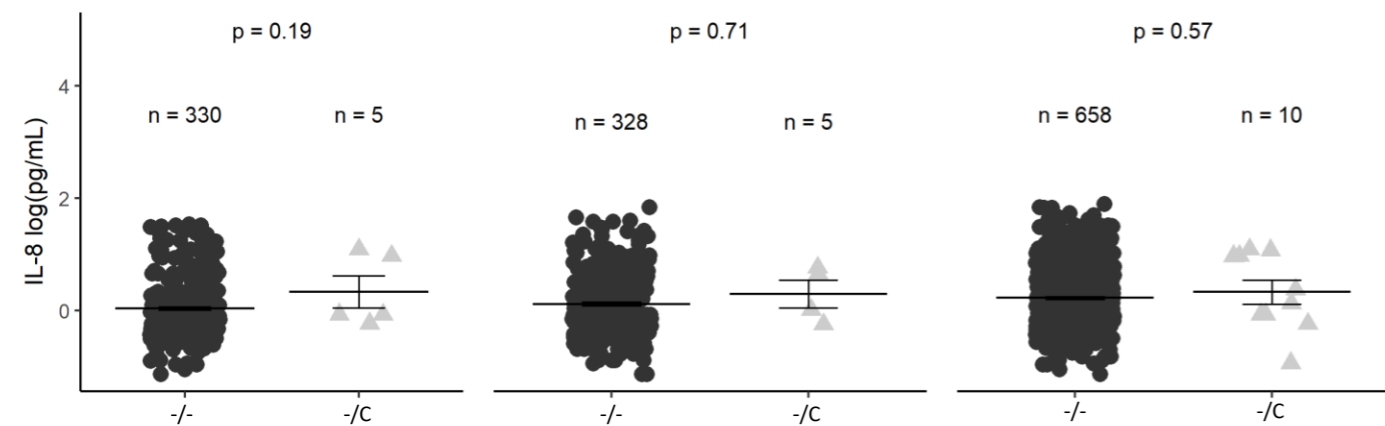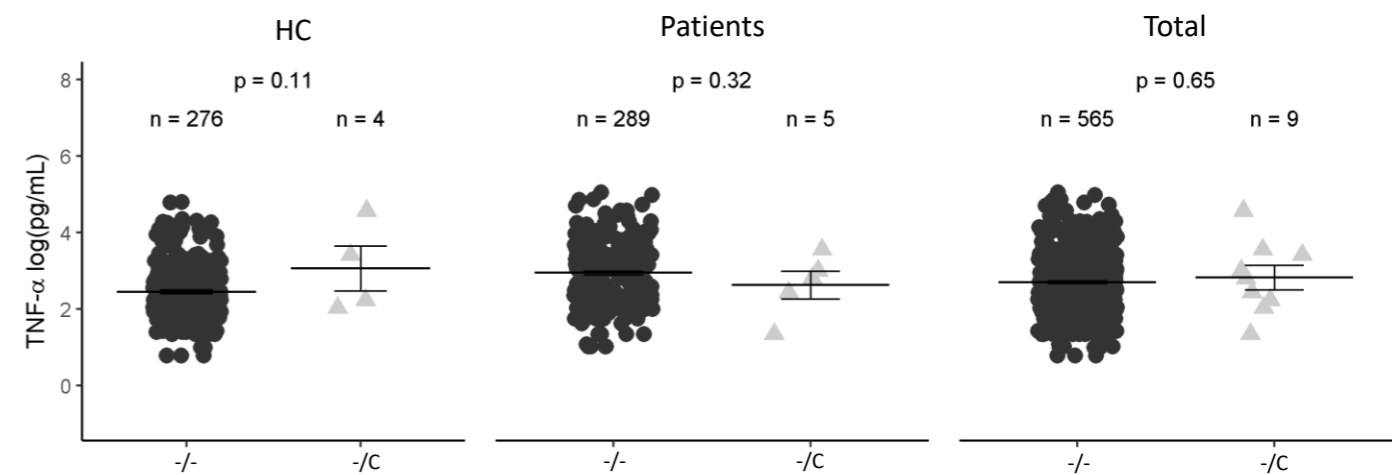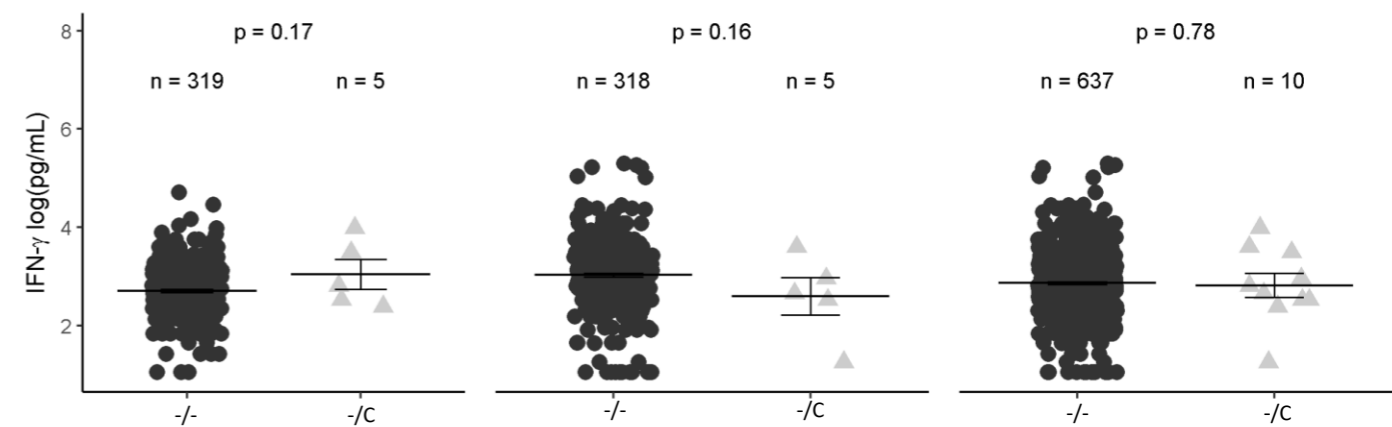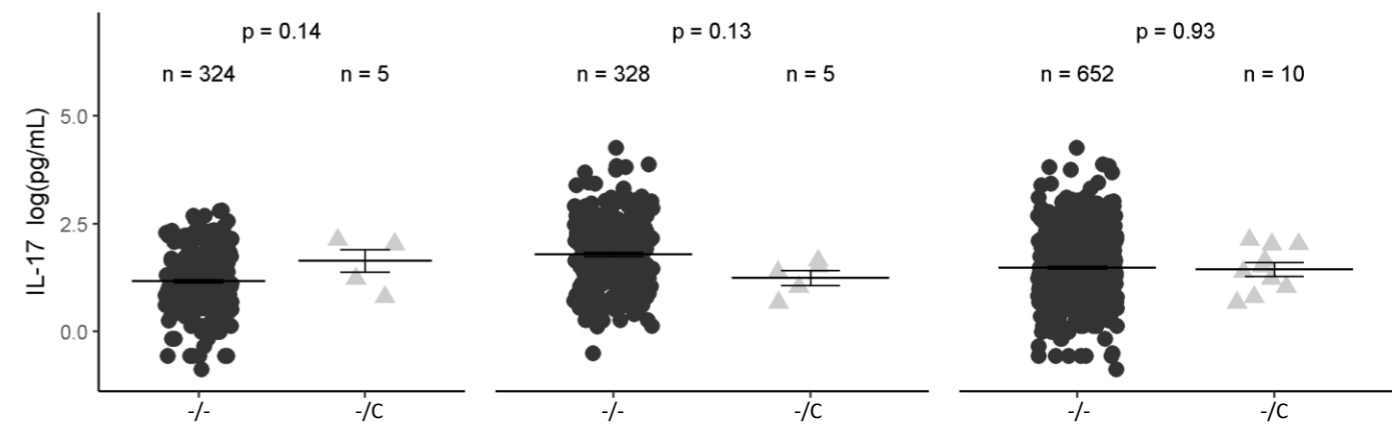

Supplement: S3 Fig — The crossbars (black) represent the mean concentrations in picogram per milliliter log-scale transformed [log(pg/mL)] and the error bars represent the standard error (SE) of means. P values < 0.05 are considered significant. (PDF) [file pone.0281814.s003.pdf]

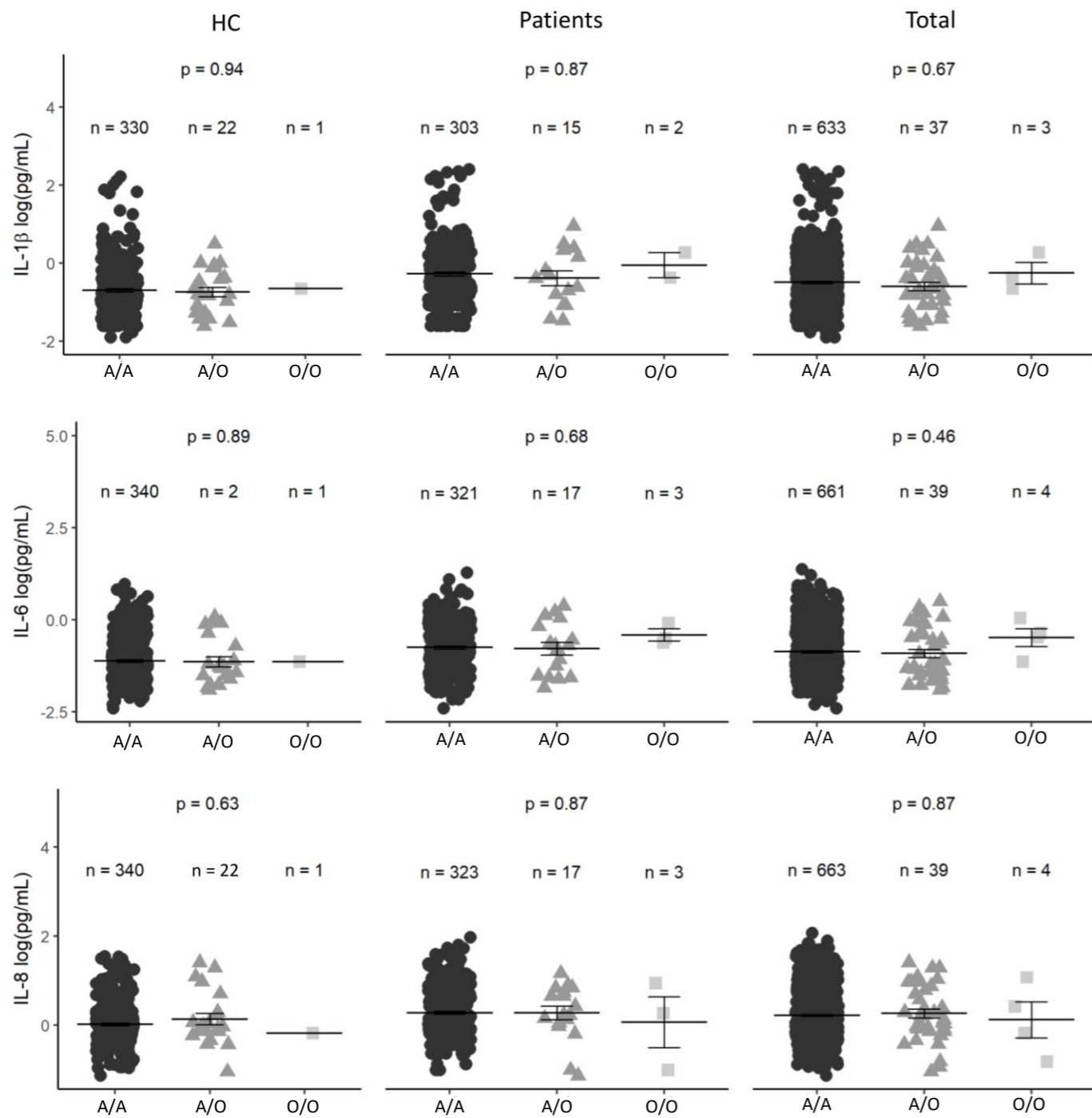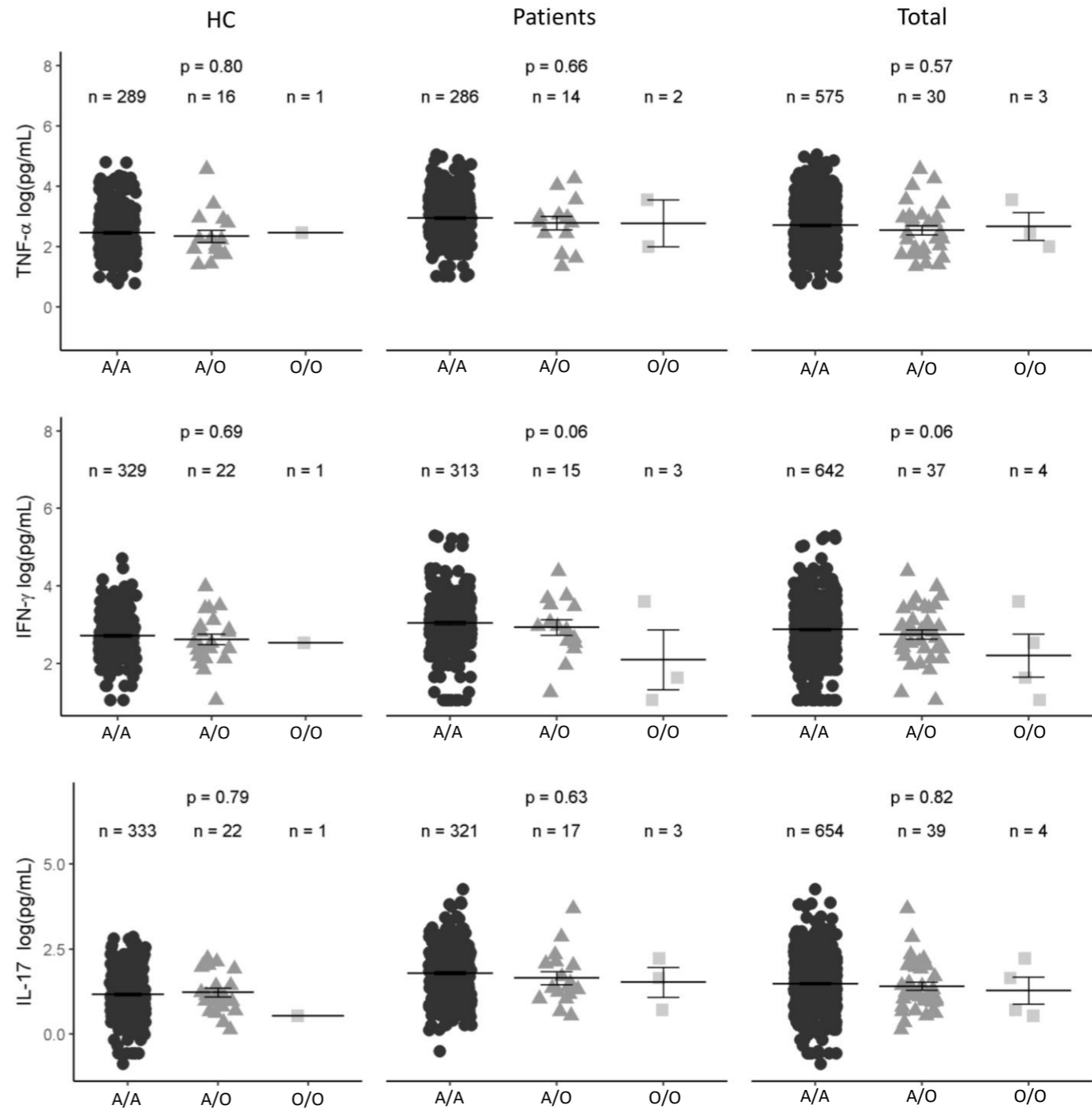

Supplement: S4 Fig — The crossbars (black) represent the mean concentrations in picogram per milliliter log-scale transformed [log(pg/mL)] and the error bars represent the standard error (SE) of means. P values < 0.05 are considered significant. The combination for all genotypes (G908R, R702W, and Lf1007ins C), common homozygous (A/A), heterozygous (A/O) and rare homozygous (O/O) are also shown in HC, Patients, and Total (A represent wild-type alleles and O mutant alleles of the three variants. (PDF) [file pone.0281814.s004.pdf]
