# Supplementary material for: Variants of NOD2 in Leishmania guyanensis-infected patients with cutaneous leishmaniasis and correlations with plasma circulating pro-inflammatory cytokines
Source: PLoS One. 2023 Feb 16;18(2):e0281814. doi: 10.1371/journal.pone.0281814 (PMC9934361; doi:10.1371/journal.pone.0281814)
Supplement: S1 Table — (DOCX) [file pone.0281814.s005.docx]

**S1 Table**. Mean levels (mean±se log pg/mL) of plasma circulating IL-8, TNF-α, IFN-γ, and IL-17 among patients with *Lg*-CL, HC, and totals (HC + Patients with *Lg*-CL) for G908R, R702W, and combinations of all genotypes.

|  | IL-8 | | |  | | TNF-α | | | |
| --- | --- | --- | --- | --- | --- | --- | --- | --- | --- |
| G908R | **G/G** | **G/C** | **C/C** | **Pv** | | **G/G** | **G/C** | **C/C** | **Pv** |
| Patients | 0.42±0.03 | 0.09±0.47 | -0.8±0.0 | 0.06 | | 2.94±0.04 | 2.69 ±0.70 | 1.99±0.0 | 0.36 |
| HC | 0.02±0.02 | -0.38±0.37 |  | 0.18 | | 2.45±0.04 | 1.56±0.17 |  | 0.14 |
| Totals | 0.21±0.02 | -0.14±0.29 | -0.8±0.0 | 0.04 | | 2.69±0.03 | 2.24±0.47 | 1.99±0.0 | 0.29 |
|  | **IFN-γ** | | | | | **IL-17** | | | |
| Patients | 3.03±0.04 | 3.31±0.54 | 1.63±0.0 | 0.10 | | 1.79±0.04 | 1.64±0.26 | 0.70±0.0 | 0.34 |
| HC | 2.71±0.02 | 2.32±0.06 |  | 0.21 | | 1.13±0.03 | 0.65±0.18 |  | 0.34 |
| Totals | 2.87±0.02 | 2.81±0.33 | 1.63±0.0 | 0.12 | | 1.45±0.03 | 1.15±0.26 | 0.70±0.0 | 0.38 |
| IFN-γ | | | | | | | | | |
| R702W | | | | | **Combination of all variants** | | | | |
|  | **C/C** | **C/T** | **T/T** | **Pv** | | **A/A** | **A/O** | **O/O** | **Pv** |
| Patients | 3.08±0.03 | 3.04±0.20 | 1.06±0.0 | 0.01 | | 3.04±0.04 | 2.93±0.19 | 2.10±0.76 | 0.06 |
| HC | 2.74±0.02 | 2.75±0.15 | 2.53±0.0 | 0.98 | | 2.71±0.02 | 2.62±0.13 | 2.53±0.0 | 0.69 |
| Totals | 2.91±0.02 | 2.88±0.12 | 1.79±0.73 | 0.06 | | 2.87±0.02 | 2.75±0.11 | 2.21±0.55 | 0.06 |
| Abbreviations: Pv: *p-*value adjusted for sex and age, and corrected for False Discovery Rates using Benjamini Hochberg. The combination for all genotypes (G908R, R702W, and Lf1007ins C), common homozygous (A/A), heterozygous (A/O) and rare homozygous (O/O) are also shown in HC, Patients, and Total (A represent wild-type alleles and O mutant alleles of the three variants). | | | | | | | | | |
